# Supplementary material for: Mineralized collagen scaffolds fabricated with amniotic membrane matrix increase osteogenesis under inflammatory conditions
Source: Regen Biomater. 2020 Apr 7;7(3):247–58. doi: 10.1093/rb/rbaa005 (PMC7266662; doi:10.1093/rb/rbaa005)
Supplement: rbaa005_Supplementary_Data [file rbaa005_supplementary_data.docx]

**Supplementary Information**

**Mineralized collagen scaffolds fabricated with amniotic membrane matrix increase osteogenesis under inflammatory conditions**

**Marley J. Dewey^1^, Eileen M. Johnson^2^, Simona T. Slater^3^, Derek J. Milner^4^,**

**Matthew B. Wheeler^2,4,5^, Brendan A.C. Harley^3,4^**

^1^ Dept. of Materials Science and Engineering

^2^ Dept. of Bioengineering

^3^ Dept. Chemical and Biomolecular Engineering

^4^ Carl R. Woese Institute for Genomic Biology

^5^ Dept. of Animal Sciences

University of Illinois at Urbana-Champaign

Urbana, IL 61801

**Supplementary Figures**


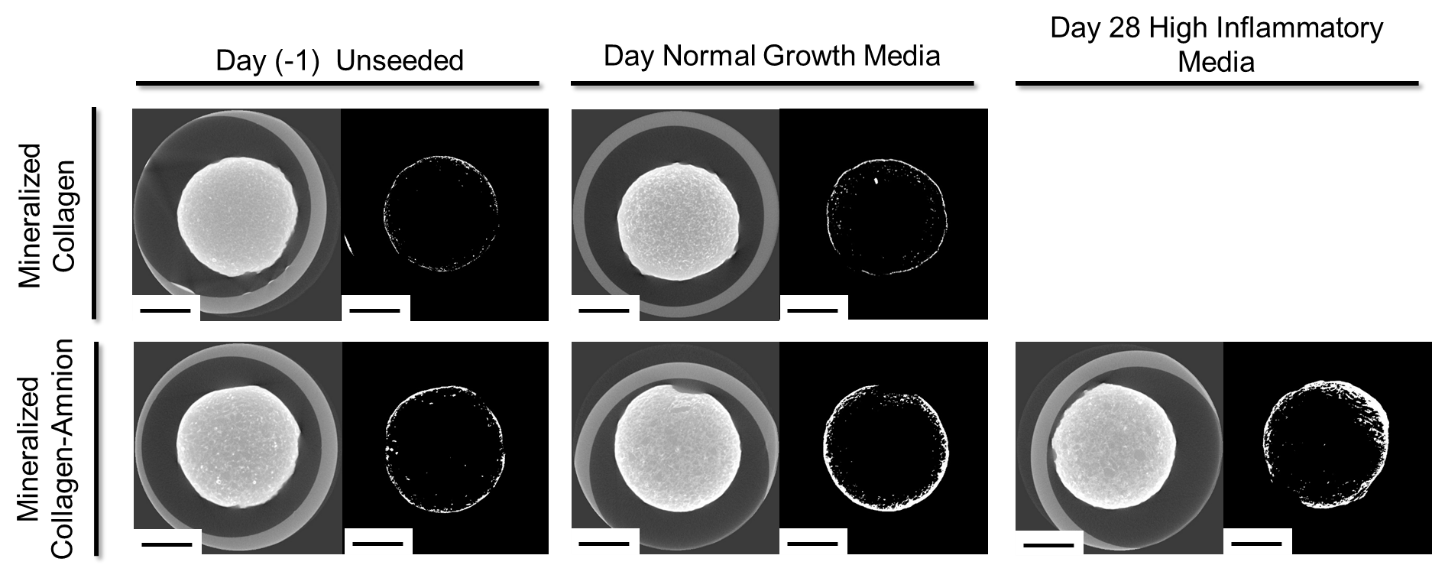


**Supplementary Figure 1. Representative Micro-CT images and ImageJ renditions of mineralized collagen and mineralized collagen-amnion scaffolds.** Raw micro-CT images presented on the left of each column and ImageJ intensified images presented on the right. Scale bar represents 2.5 mm.


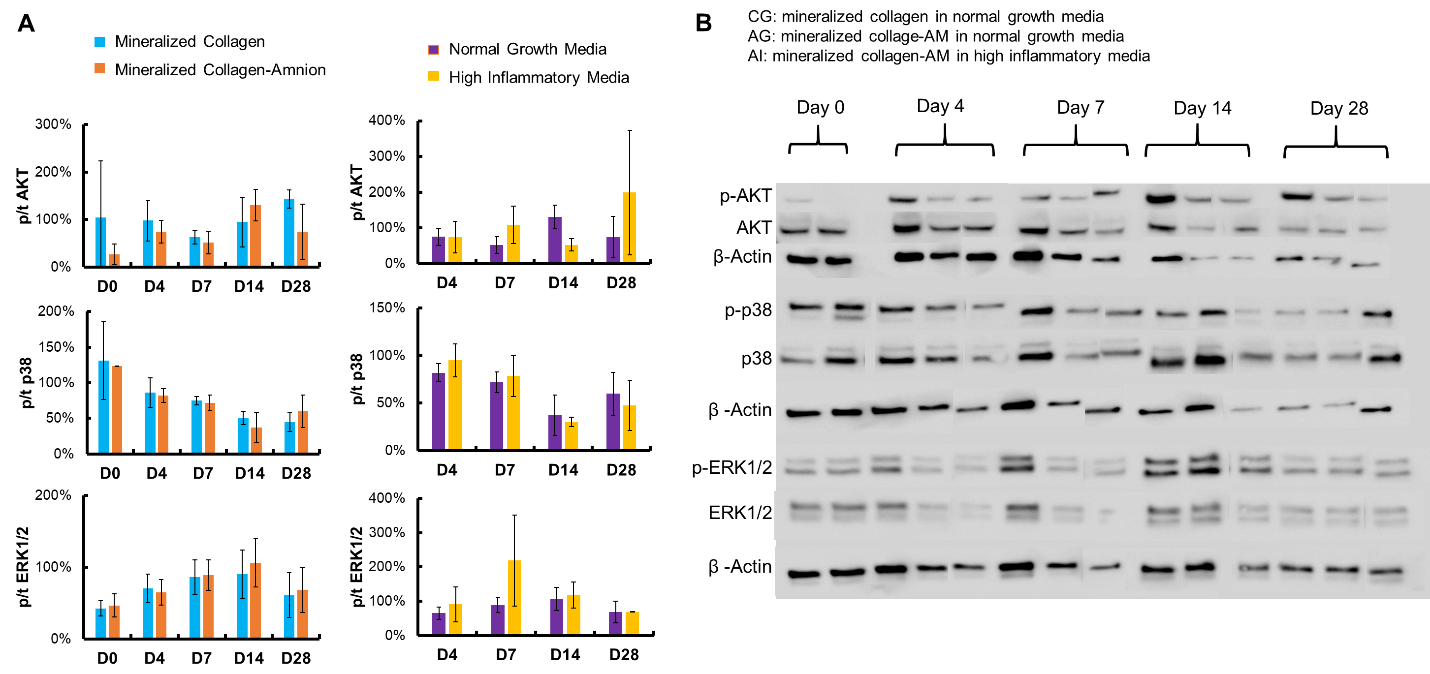


**Supplementary Figure 2. Protein activity of mineralized collagen scaffolds compared to mineralized collagen-amnion scaffolds in normal and high inflammatory media.** Osteogenic protein activity was quantified with Western Blots. (A) Phosphorylated over total protein activity in scaffolds. No significance (p < 0.05) was observed between any groups. Data expressed as mean ± standard deviation (n= 3). (B) Representative western blot images with β-actin as a control.


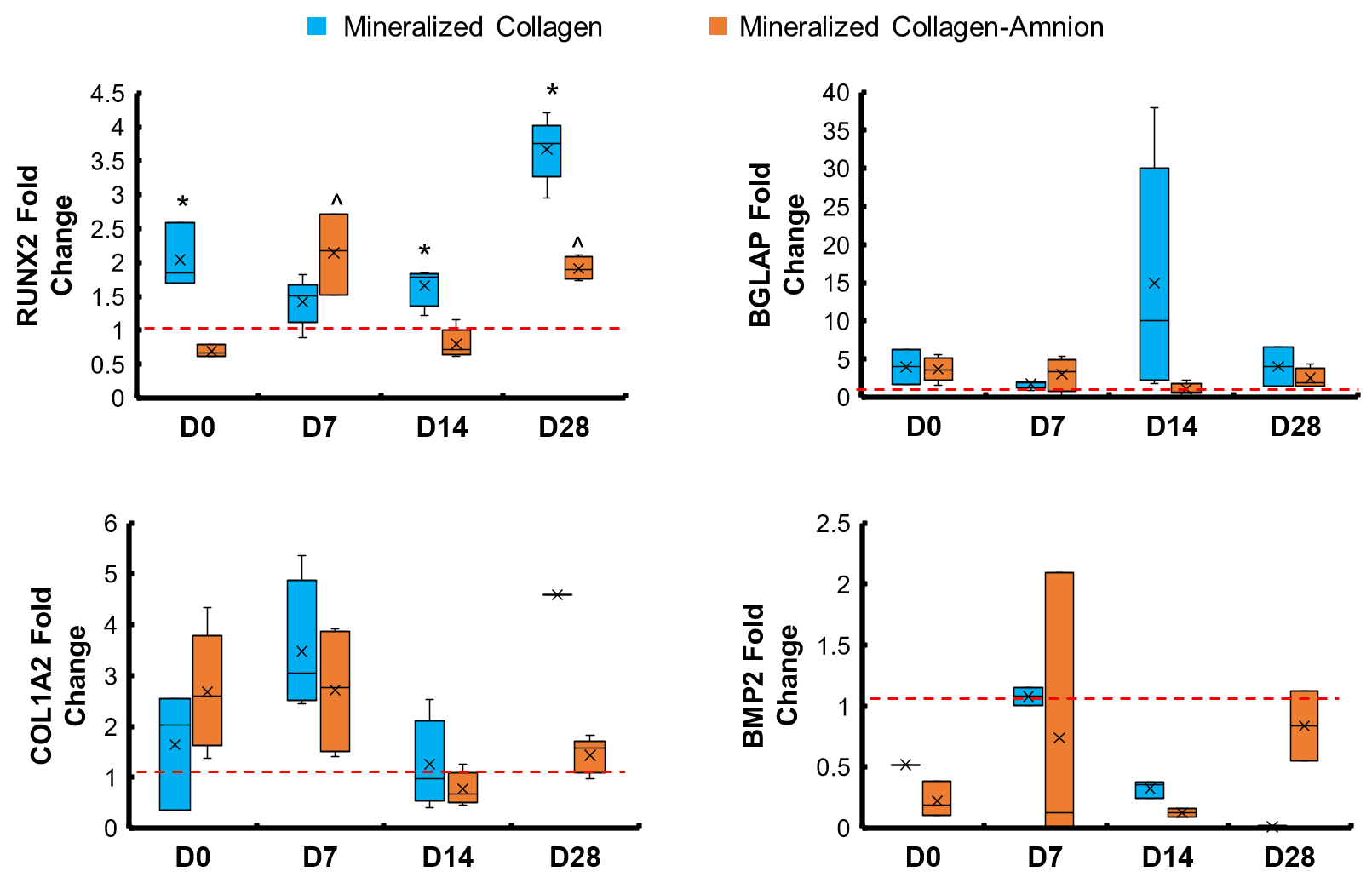


**Supplementary Figure 3. Osteogenic gene expression of mineralized collagen and mineralized collagen-amnion scaffolds in normal growth media.** Gene expression was evaluated by RT-PCR and normalized to the expression of cells before seeding on scaffolds. Below the graphs are brief statements about each gene of interest. * indicates the mineralized collagen scaffold was significantly (p < 0.05) greater than the mineralized collagen-amnion scaffold on the same day. ^ indicates one scaffold type was significantly (p < 0.05) greater than the same type compared to day 0. Data expressed as mean ± standard deviation (n=5).


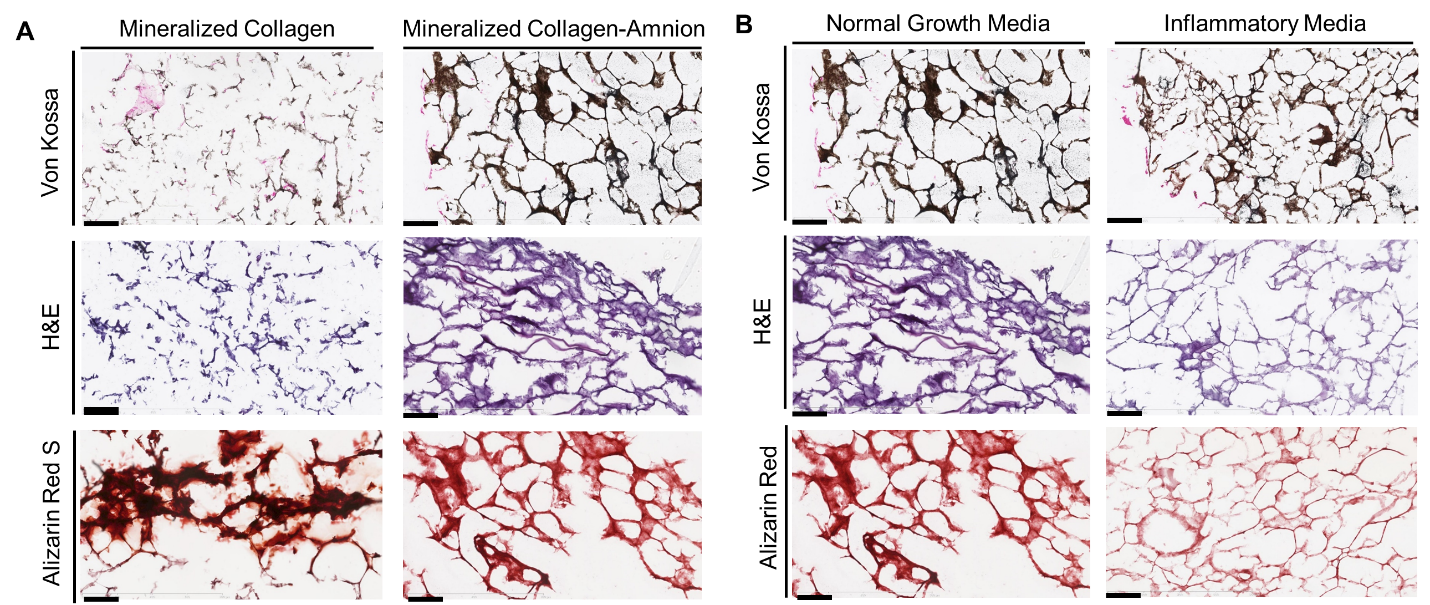


**Supplementary Figure 4. Histological staining of mineralized collagen-amnion scaffolds in normal growth media and inflammatory media after 28 days.** (A) Mineralized collagen and mineralized collagen-amnion scaffolds in normal growth media. (B) Mineralized collagen-amnion scaffolds in normal growth media and inflammatory media. Scale bar represents 200µm.


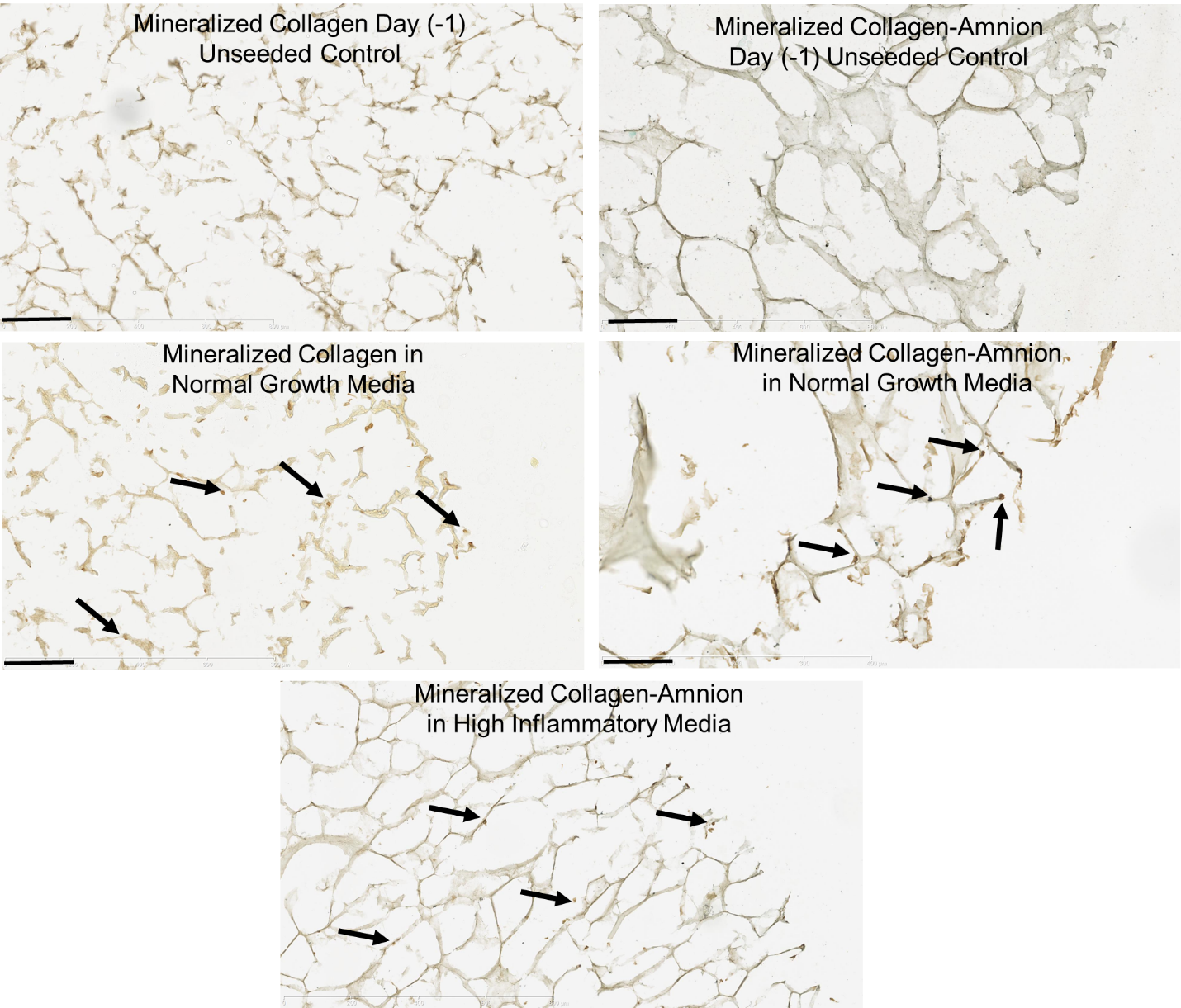


**Supplementary Figure 5. Osteopontin-stained mineralized collagen and mineralized collagen-amnion scaffolds.** Scaffolds were stained using immunohistochemical procedures. Black arrows represent OPN positive cells. Scale bars represent 200 µm.
